# PIKfyve Regulates the Endosomal Localization of CpG Oligodeoxynucleotides to Elicit TLR9-Dependent Cellular Responses

**DOI:** 10.1371/journal.pone.0073894

**Published:** 2013-09-09

**Authors:** Kaoru Hazeki, Masami Uehara, Kiyomi Nigorikawa, Osamu Hazeki

**Affiliations:** Division of Molecular Medical Science, Graduate School of Biomedical Sciences, Hiroshima University, Hiroshima, Japan; Indiana University School of Medicine, United States of America

## Abstract

TLR9 is a receptor for oligodeoxynucleotides that contain unmethylated CpG motifs (CpG). Because TLR9 resides in the endoplasmic reticulum during the quiescence state, CpG binding to TLR9 requires membrane trafficking, which includes the maturation of the CpG-containing endosome. In the present study, we examined the role of PIKfyve, a phosphatidylinositol 3-phosphate 5-kinase, in the regulation of TLR9 signaling. The PIKfyve inhibitor YM201636 inhibited co-localization of the CpG-containing endosome with LysoTracker, which stains acidic organelle, and with TLR9. YM201636 increased the co-localization of CpG with the early endosome marker EEA1 but decreased co-localization with the late endosome marker LAMP1. Similar results were obtained in Raw264.7 cells containing shRNA that targets PIKfyve. CpG-mediated phosphorylation but not lipopolysaccharide (LPS)-mediated phosphorylation of IKK, p38 MAPK, JNK and Stat3 was severely impaired by the loss of PIKfyve function. CpG-mediated expression of cytokine mRNA was also decreased in the absence of PIKfyve. These findings demonstrate a novel role of PIKfyve in TLR9 signaling.

## Introduction

TLRs recognize specific molecular patterns expressed by microorganisms, which results in host immune responses against pathogens. TLRs can be divided into two groups based on their subcellular localization. TLR 1, 2, 4, 5 and 6 are cell surface receptors and recognize surface structures of pathogens, whereas TLR 3, 7, 8 and 9 reside in intracellular compartments and detect microbial nucleic acids [1]. TLR9 is a receptor for oligodeoxynucleotides that contain unmethylated CpG motifs (CpG). Because TLR9 possesses retention signals to keep it in the endoplasmic reticulum, TLR9 cleavage by proteases is necessary for its recruitment to the endosomes where the receptor meets its ligand [2–4]. CpG has been shown to associate with TLR9 through internalization. Thus, intracellular trafficking of both the receptor and the ligand is essential in TLR9 signaling [5,6].

Phosphoinositides (PIs), which are phosphorylated derivatives of phosphatidylinositol (PtdIns), have central roles in cytoskeletal dynamics and membrane trafficking [7]. One of the most well documented PIs associated with membrane trafficking is PtdIns(3) P, which is mainly produced by the class III PI3-kinase Vps34. PtdIns(3) P and Vps34 are enriched in subcellular compartments such as the phagosome, autophagosome and early endosome. Previous studies have shown that Vps34 is involved in TLR9 signaling by regulating CpG uptake [8]. In addition, studies have suggested that ADP-ribosylation factor 6 (ARF6) is involved in this Vps34 function [9]. However, the effect of newly synthesized PtdIns(3) P on the endosome has not been previously described. Schematically, PtdIns(3) P is hydrolyzed to form PtdIns and is also phosphorylated to form PtdIns(3,5) P_2_ and PtdIns(3,4) P_2_.

PtdIns(3) P 5-kinase, known as PIKfyve in mammals and Fab1 in yeast, largely synthesizes PtdIns(3,5) P_2_ from PtdIns(3) P on the late endosome [10]. Compared with other PtdInsPs, the role of PtdIns(3,5) P_2_ is unclear because of its low abundance [10]. PIKfyve knockout or transgenic mouse models have not been available because of embryonic lethality [11]; however, experiments utilizing siRNA knockdown and the pharmacological inhibitor YM201636 have revealed that the inhibition of this lipid kinase activity results in enlarged endolysosome formation and defective endosome-TGN trafficking [12–14]. It is also known that PIKfyve-dependent fusion of macropinosome-late endosome/lysosome is required for proper maturation of *Salmonella* -containing vacuole and replication of the pathogen [15]. Hence, we hypothesize that CpG does not associate with TLR9 in the absence of PIKfyve. To demonstrate this hypothesis, we used the specific inhibitor YM201636. In addition, we cloned PIKfyve-deficient Raw264.7 cells that were stably transfected with shRNA targeting the lipid kinase. Both the pharmacological and the knockdown strategies showed that PIKfyve contributes to the localization of CpG and to the resultant activation of signaling molecules. CpG-induced cytokine production was ultimately decreased by the deficiency in PIKfyve function. The results of the present study also suggested that PIKfyve may regulate the localization of other TLRs and their ligands, which may influence the direction of innate immunity.

## Materials and Methods

### Reagents

Labeled (5’-rhodamine and 5’-FITC) and unlabeled CpG DNA-B1668 (HPLC-purified phosphorothioate with the sequence of TCC ATG ACG TTC CTG ATG CT) were synthesized by Hokkaido System Science (Sapporo, Japan). Rox-labeled CpG with the same sequence was kindly donated by Taniguchi, T (University of Tokyo). LPS (*E. coli* serotype 0111: B4) was obtained from Sigma-Aldrich. LysoTracker Red was acquired from Lonza. The Protein Assay Kit was purchased from Bio-Rad. YM201636 was obtained from CALBIOCHEM. R848 and Malp2 were purchased from ENZO life sciences. Anti-PIKfyve was obtained from Abnova, anti-EEA1 was obtained from GenScript, anti-LAMP1 was obtained from BioLegend, anti-pIKK, -pp38, -pJNK, -pStat3 and -pERK were all obtained from Cell Signaling. The IL-10 and IL-12p40 ELISA assay kit was purchased from Biolegend. The TNF-α assay kit was purchased from PEPROTECH.

### Animals and cell isolation

All animal experiments were carried out in accordance with the NIH Guide for Care and Use of Laboratory Animals and approved by the animal care and use committee at Hiroshima University (Permit number: A10-86).

Female C57BL/6 mice, 8–12 weeks old, were purchased from Japan SLC, Inc. Thioglycollate-elicited macrophages were harvested from these mice. Mice were injected intra-peritoneally with 3 mL of 3% thioglycollate broth. After 3 days, the peritoneal exudate cells were collected by washing the peritoneal cavity with ice-cold phosphate-buffered saline (PBS). The cells were seeded at approximately 5-10x 10^5^ cells/well in 24-well plates and incubated in humidified 5% CO_2_ at 37 °C for 1–2 h in RPMI 1640 medium supplemented with 10% fetal bovine serum (FBS). These conditions enabled the cells to adhere to the wells. The non-adherent cells were removed by washing with PBS and the attached cells were used for experiments. Cos7 cells were cultured in DMEM medium supplemented with 10% FBS.

### Cells lacking PIKfyve

Raw264.7 cells (ATCC) lacking PIKfyve were prepared as follows: Oligonucleotides with sequences targeting the protein were cloned into the pH1 vector downstream of the H1 RNA promoter as described [16,17] to express siRNA (small interfering RNA) hairpins. A pair of oligonucleotides were synthesized (Hokkaido Life Sciences, Sapporo, Japan) with the following sequences: 5′-CCC(X) _19_ TTCAAGAGA(Y) _19_ TTTTTGGAAA-3′ and 5′-CTAGTTTCCAAAAA(Y) _19_ TCTCTTGAA(X) _19_ GGGTGCA-3′, where (X)_19_ is the coding sequence and (Y)_19_ is the complementary sequence. The oligonucleotide pair was annealed and ligated downstream of the H1 RNA promoter at the PstI and XbaI sites of the pH1 vector. The vectors were transfected into Raw264.7 cells [(5-10) × 10^6^ cells] at 250 V/950 µF (Gene Pulser II; Bio-Rad). At 24 h after transfection, puromycin (3 µg/ml) was added to the cells, and the incubation was continued to select for resistant cells. Control cells were prepared as described above with the pH1 vector containing a 400-bp stuffer sequence instead of the target sequence.

### Transfection of EGFP-TLR9

EGFP-TLR9 was kindly donated by Miyake K. (University of Tokyo) [18]. Raw264.7 cells were maintained in RPMI 1640 medium containing 4.5 g/l glucose and 10% FCS at 37°C in a humidified 5% CO_2_ atmosphere. EGFP-TLR9 was transfected by the Neon^TM^ transfection system (Invitrogen) according to the manufacturer’s protocol. Cells were used for microscopic analysis 24-h after the transfection.

### Microscopy

Raw264.7 cells in multi-well, glass-bottom dishes (Greiner bio-one) were incubated with LysoTracker Red in the presence or absence of YM201636 for 30 min, added with FITC-CpG, incubated for additional 10 min and finally washed vigorously five times with PBS by pipetting up and down to remove CpG in the medium. Alternatively, Raw264.7 cells transfected with EGFP-TLR9 were incubated with YM201636 for 30 min, added with ROX-CpG, incubated for additional 10 min and washed. The translocation of CpG within the cells was chased up to 30 min at 37°C by time-lapse imaging. For immunostaining, cells were treated with or without YM201636, added with CpG, washed with PBS, and incubated for the indicated periods before being fixed with PBS containing 4% formaldehyde for 15 min. The cells were permeabilized with PBS containing 0.3% Triton X-100 and 0.5% BSA for 60 min, and incubated with anti-LAMP1 or anti-EEA1 at 4 °C overnight. The cells were then incubated with Alexa 647-labeled goat anti-mouse IgG antibody (Fab’)_2_ for 2 h at room temperature. Microscopic studies were performed using the Keyence BZ-9000 with CFI Plan Apo VC60xH lens (Keyence, Osaka, Japan). For quantitative analysis, with the exception of the quantification of time-laps imaging, Z-stacks were captured at 1 µm steps over a Z-axis distance of 5 µm (Figure S1). Stacks were reconstructed and analyzed by BZ-H2C (Keyence application for BZ-9000), which allowed us to determine the fluorescent area. The quantification condition was saved and applied for the all images within the figures. We analyzed several stacked images; each contained 250-300 cells, to get "co-localization index" which was calculated as (merged area)/(total CpG area) x 100. The data from at least 4 images are shown as the mean ± SD. Significant differences were determined at the level of p < 0.05 or 0.01 with Student’s t test. For illustration, the images were contrast enhanced, pseudocolored, merged, cropped and assembled.

### Western blot

Cells were washed with PBS and lysed in 50 µL of lysis buffer containing 25 mM Tris-HCl (pH 7.4), 0.5% Nonidet P-40, 150 mM NaCl, 1 mM sodium orthovanadate (Na _3_VO_4_), 1 mM EDTA, 0.1% BSA, 20 mM sodium fluoride, 1 mM phenylmethylsulfonyl fluoride, 2 µM leupeptin, 20 µM p-amidinophenylmethylsulfonyl fluoride, and 1 mM dithiothreitol. The cell lysates were centrifuged at 15,000 rpm for 10 min. Supernatants were collected, and the protein concentration was determined using the Bio-Rad assay kit. Total cell lysates (100 µg protein) were mixed with 10 µL of 5x sample buffer (62.5 mM Tris (pH 6.8), 1% SDS, 10% glycerol, 5% 2-mercaptoethanol, and 0.02% bromophenol blue) and heated at 100 °C for 5 min. The proteins were separated by SDS-PAGE and transferred electrophoretically onto a polyvinylidene difluoride (PVDF) membrane (Millipore). The membrane was blocked with 5% skim milk and incubated with the appropriate antibodies. Antibody binding was detected using a chemiluminescent substrate (PerkinElmer).

### RT-PCR

Total RNA was isolated using Sepasol RNA I (Nacalai tesque, Kyoto, Japan). Single-stranded cDNA was synthesized with M-MLV reverse transcriptase. The cytokine cDNAs were amplified by a PCR method using the specific primers provided in Table 1.

**Table 1:** PCR Primers.

Target gene	primer	sequence	position	product	annealing
IL-1β	IL-1β (+) IL-1β (-)	caggatgaggacatgagcacc ctctgcagactcaaactccac	330-350 776-756	447 bp	50°C
IL-10	IL-10 (+) IL-10 (-)	ctcttactgactggcatgaggatc ctatgcagttgatgaagatgtcaaatt	98-121 572-546	475 bp	62°C
IL-12p40	IL-12p40 (+) IL-12p40 (-)	gtagaggtggactggactcc gcagacagagacgccattcc	259-278 683-664	425 bp	55°C
GAPDH	GAPDH (+) GAPDH (-)	aacgaccccttcattgac tccacgacatactcagcac	169-186 359-341	191 bp	55°C

## Results

### PIKfyve inhibition resulted in swollen vacuole formation in Raw264.7 cells

**Figure 1:**
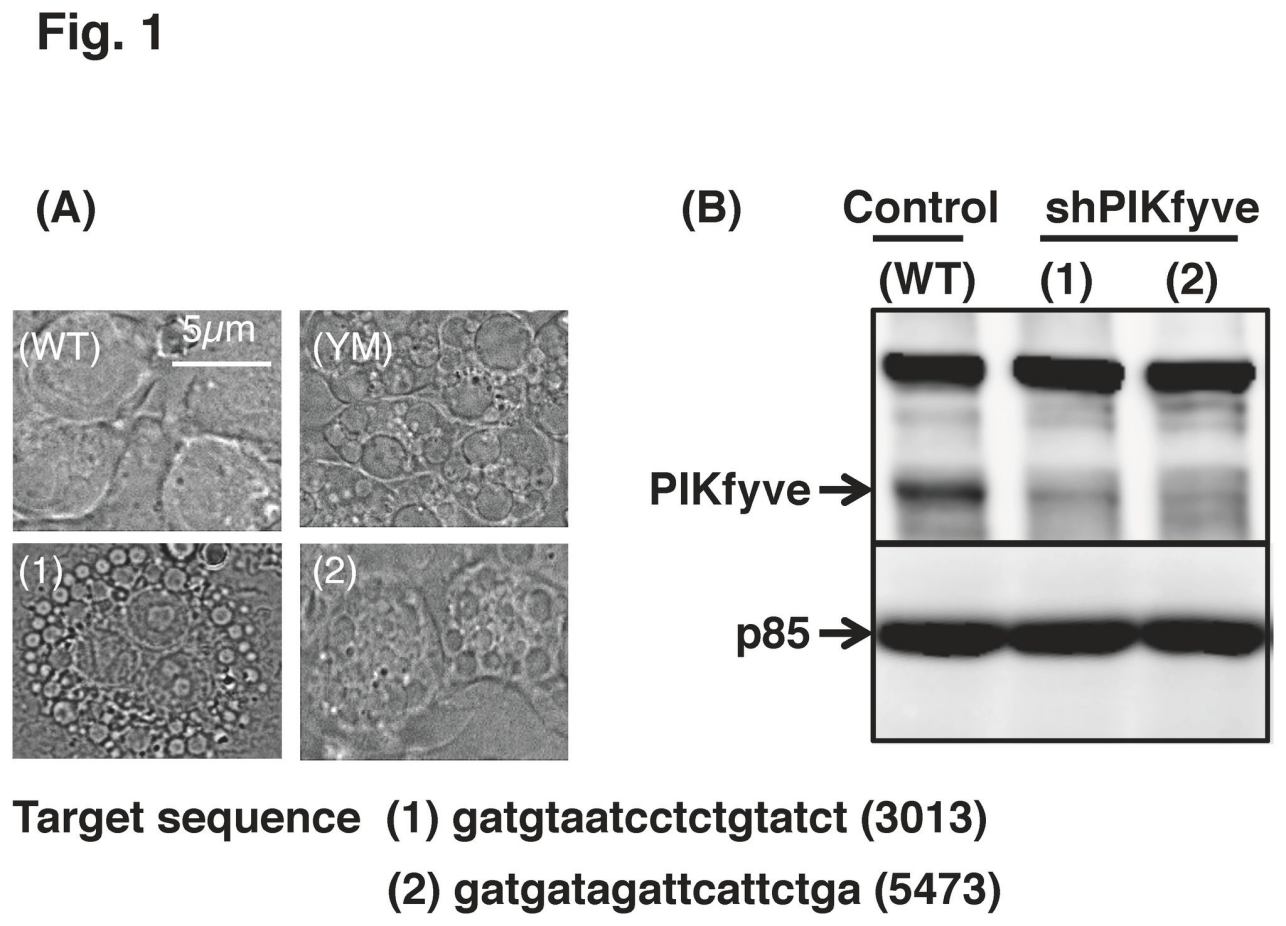
PIKfyve inhibition resulted in swollen vacuole formation in Raw264.7 cells. Wild type Raw 264.7 cells (WT, YM) were incubated with (YM) or without (WT) 1 µM YM201636 for 30 min. Two lines of Raw 264.7 cells deficient in PIKfyve (shPIKfyve-1, and shPIKfyve-2) were prepared with the respective target sequences (1,2) shown under the images. (A) Phase contrast images are shown. (B) The expression of the target protein (PIKfyve) and the invariable control (PI3K p85) was estimated by Western blotting.

Treatment of Raw264.7 cells with a PIKfyve inhibitor YM201636 resulted in the formation of swollen vacuoles that were visible by phase contrast light microscopy (Figure 1A). A similar morphological change has been observed in HeLa cells [13], and is reported to result from the abated levels of PtdIns(3,5) P_2_ and the resultant disruption of endosome maturation [10]. We then prepared two lines of PIKfyve-deficient Raw264.7 cells (referred to as shPIKfyve-1 and shPIKfyve-2 cells; Figure 1B) by introducing shRNAs that target different sequences in PIKfyve mRNA. Knockdown of PIKfyve resulted in the formation of swollen vacuoles (Figure 1A).

### The PIKfyve inhibitor prevented the endosomal translocation of CpG

**Figure 2:**
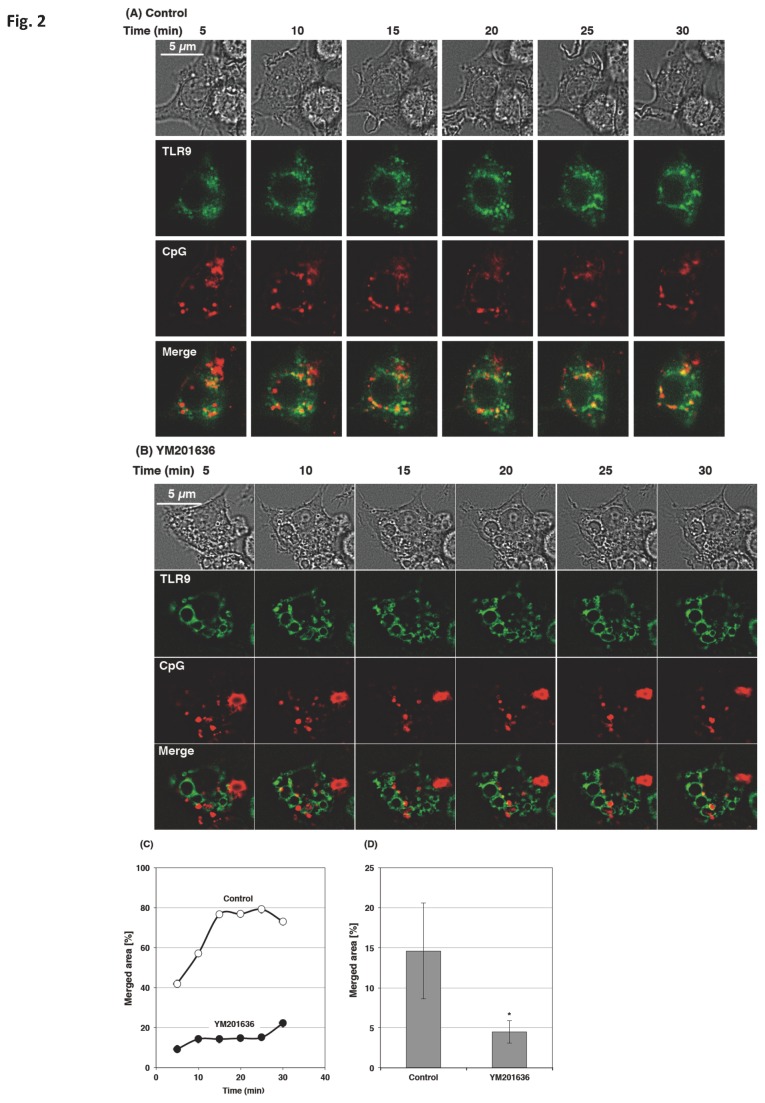
The PIKfyve inhibitor prevented co-localization of CpG with TLR9. (A–D) Raw264.7 cells transfected with EGFP-TLR9 were incubated with or without 1 µM YM201636 for 30 min, added with 3 µM ROX-CpG, incubated for additional 10 min, and then washed. (A, B) The translocation of CpG within the cells was chased in the presence (B) or absence (A) of 1 µM YM201636 up to 30 min at 37°C. (C) The cells shown in (A) and (B) were analyzed by a quantification software (BZ-H2C) and plotted. (D) After the wash, the cells were further incubated at 37 °C in the presence or absence of YM201636 for 30 min. Z-stacks were captured at 1 µm steps over a Z-axis distance of 5 µm. Stacks were reconstructed and analyzed by the software. We analyzed 6 stacked images; each contained 250-300 cells, to estimate the merged area. * p<0.05.

Raw264.7 cells transfected with EGFP-TLR9 were incubated with ROX-CpG for 10 min, washed five times, and the fluorescence were chased up to 30 min (Figure 2A, B). Co-localization of CpG with TLR9 was estimated by determining the merged area (Figure 2C). Although the intensity of total CpG fluorescence decreased gradually with the time, the merged area increased and reached a plateau level at 15 min, which was maintained about 30 min after the addition of CpG (Figure 2C). In the cells treated with YM201636, the CpG co-localization with TLR9 was decreased throughout the 30-min chase periods (Figure 2C). CpG began to co-localize with TLR9 at 30 min in these cells (Figure 2C). In Figure 2D, the merged area after the 30-min chase period were quantified in 6 separate images (each contained 250-300 cells).

**Figure 3:**
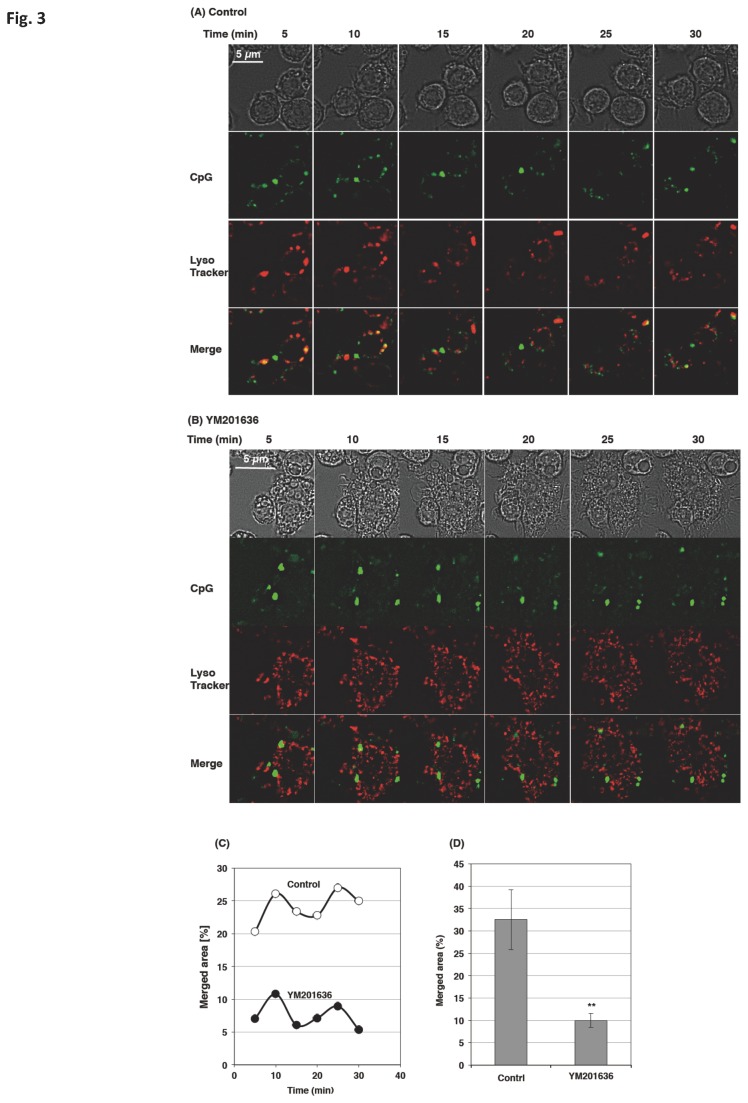
PIKfyve inhibitor interfered with the co-localization of the CpG-containing endosome and LysoTracker. (A–D) Raw264.7 cells were incubated with 50 nM LysoTracker in the absence or presence of 1 µM YM201636 for 30 min, added with 3 µM FITC-CpG, incubated for additional 10 min, and then washed. (A, B) The translocation of CpG within the cells was chased in the presence (B) or absence (A) of 1 µM YM201636 up to 30 min at 37°C. (C) The cells shown in (A) and (B) were analyzed by a quantification software (BZ-H2C) and plotted. (D) Immediately after the wash, Z-stacks were captured at 1 µm steps over a Z-axis distance of 5 µm. Stacks were reconstructed and analyzed by the software. We analyzed 6 stacked images; each contained 250-300 cells, to estimate the merged area. ** p<0.01.

Because CpG is transferred to late endosome to associate with TLR9 [5], we next investigated the co-localization of the CpG-containing endosome with LysoTracker Red, which is known to stain acidic organelles. The cells were first loaded with LysoTracker for 30 min, added with CpG and vigorously washed. CpG co-localization with LysoTracker is observable in normal cells but not in the YM201636-treated cells (Figure 3A, B). The co-localization peaked at the beginning of the chase and almost unchanged up to 30 min (Figure 3C). Statistical analysis confirmed that the co-localization of CpG-containing endosome with LysoTracker was severely inhibited by YM201636 (Figure 3D).

**Figure 4:**
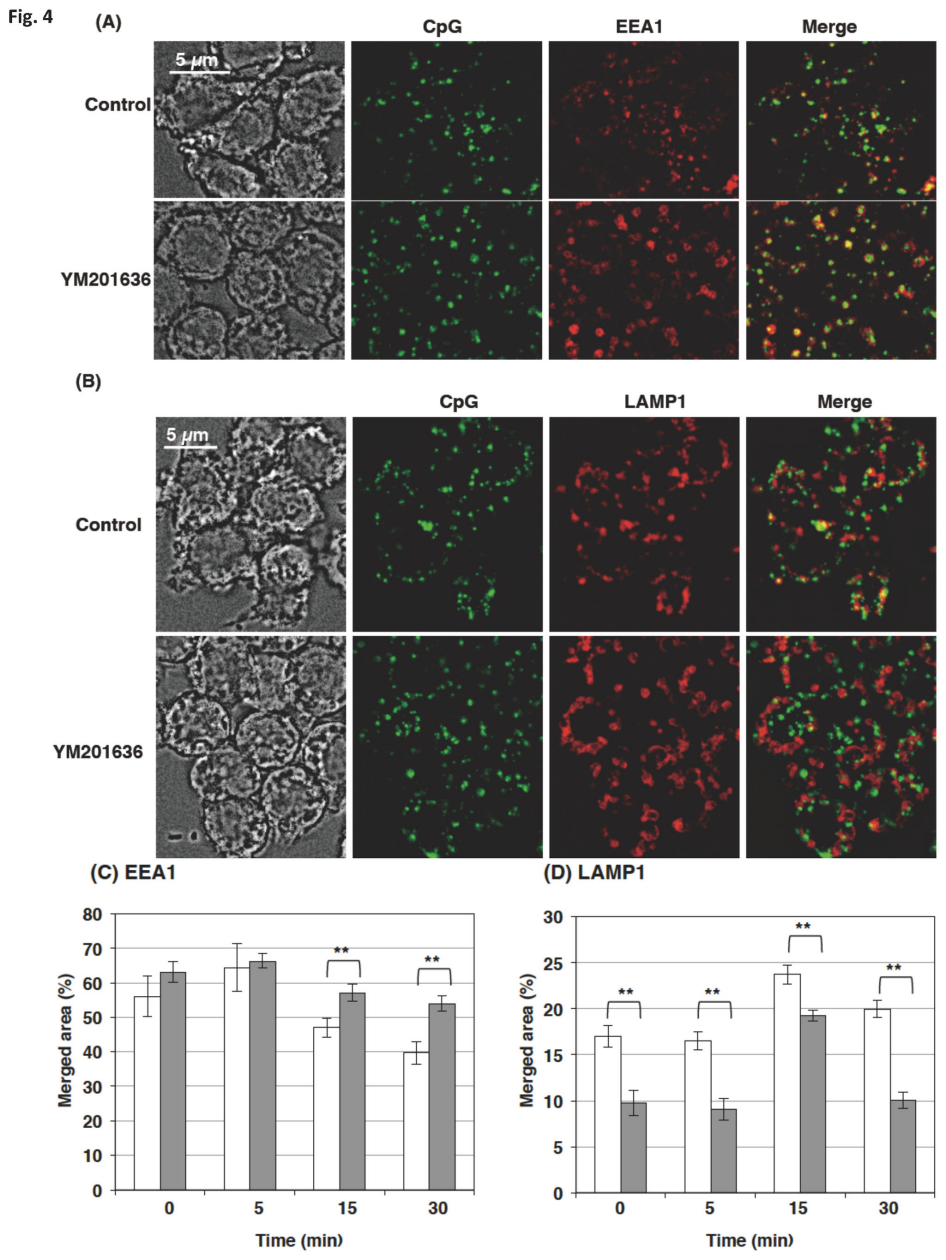
PIKfyve inhibitor prevented the endosomal translocation of CpG. (A–D) Raw264.7 cells were incubated with (gray bars in C, D) or without (white bars in C, D) 1 µM YM201636 for 30 min, added with 3 µM FITC-CpG, incubated for additional 10 min, and then washed. The translocation of CpG in the cells was chased for 30 min (A, B) or for the indicated times (C, D) in the presence or absence of 1 µM YM201636. The cells were washed, fixed, permeabilized, and stained with anti-EEA1 antibody (A, C) or anti-LAMP1 antibody (B, D) and Alexa 647-labeled secondary antibody. Z-stacks were captured at 1 µm steps over a Z-axis distance of 5 µm. (A, B) Stacked images are shown. (C) The images were analyzed by a quantification software (BZ-H2C). We analyzed 6 stacked images; each contained 250-300 cells, to estimate the merged area. ** p<0.01.

The effect of YM201636 on CpG co-localization with endosome markers, EEA1 and LAMP1, was next studied. The cells treated with or without YM201636 were incubated with CpG and then fixed with paraformaldehyde before being stained for the endosome markers. The effect of YM201636 on the CpG co-localization with the early endosome marker EEA1 was not prominent (Figure 4A). However, a detailed analysis by pulse-chase technique indicated that YM201636 increased the amount of CpG that resides in the early endosome at 15 min (Time 15 in Figure 4C) after a 10-min pulse period (Time 0 in Figure 4C). YM201636 inhibited markedly the CpG co-localization with the late endosome marker LAMP1 (Figure 4B). Pulse-chase experiments indicated that the CpG localization with LAMP1 peaked at 15 min after the removal of CpG (Figure 4D).

YM201636 is reported to inhibit PI 3-kinase at higher concentrations [19]. Since PI 3-kinase has been implicated in trafficking of CpG-containing vesicles [20], the above effects of this inhibitor might be attributable to its effect on PI 3-kinase. The possibility prompted us to examine the dose-dependence of the YM201636 action on the PI 3-kinase activity (Figure S2). YM201636 inhibited partly the PI 3-kinase activity at 3 µM, but has little effect at 1 µM. The results in Figures 1-4 were obtained at 1 µM of the inhibitor, suggesting that the PI 3-kinase inhibition may be a minor, if any, mechanism of the YM201636 action on the CpG localization. We thus next used the PIKfyve knockdown cells (Figure 1B) to confirm the role of PIKfyve in the CpG translocation.

### The knockdown of PIKfyve impaired the endosomal translocation of CpG

**Figure 5:**
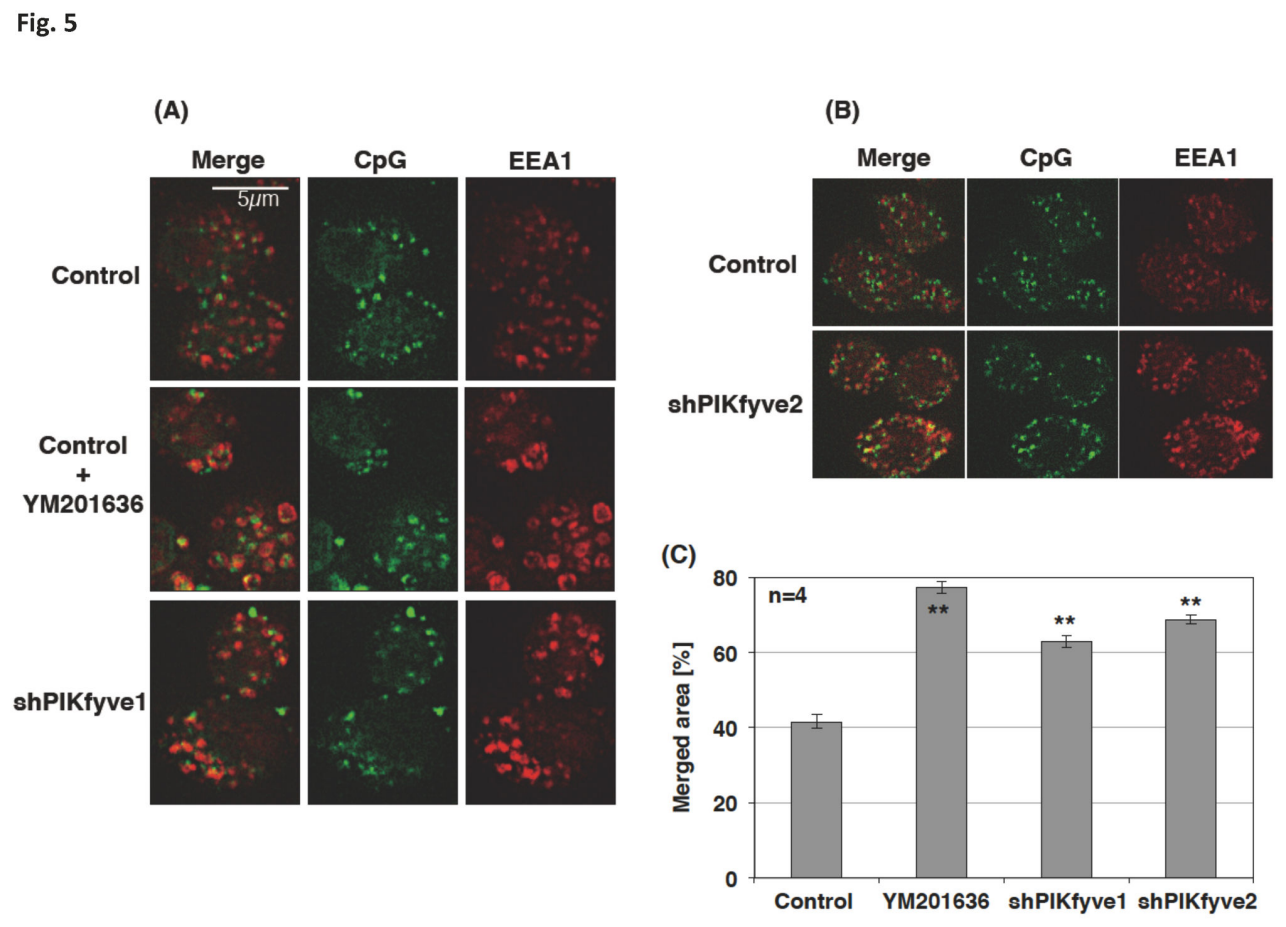
CpG preferentially stayed in the early endosome and in PIKfyve-deficient cells. Control cells, cells treated with 1 µM YM201636 for 30 min, or PIKfyve-deficient cells (shPIKfyve-1 or shPIKfyve-2 cells) were incubated with 0.5 µM FITC-CpG for 10 min. The cells were washed, fixed, permeabilized, and stained with anti-EEA1 antibody and Alexa 647-labeled secondary antibody. Z-stacks were captured at 1 µm steps over a Z-axis distance of 5 µm. (A, B) Stacked images are shown. (C) The images were analyzed by a quantification software (BZ-H2C). We analyzed 4 stacked images; each contained 250-300 cells, to estimate the merged area. ** p<0.01.

**Figure 6:**
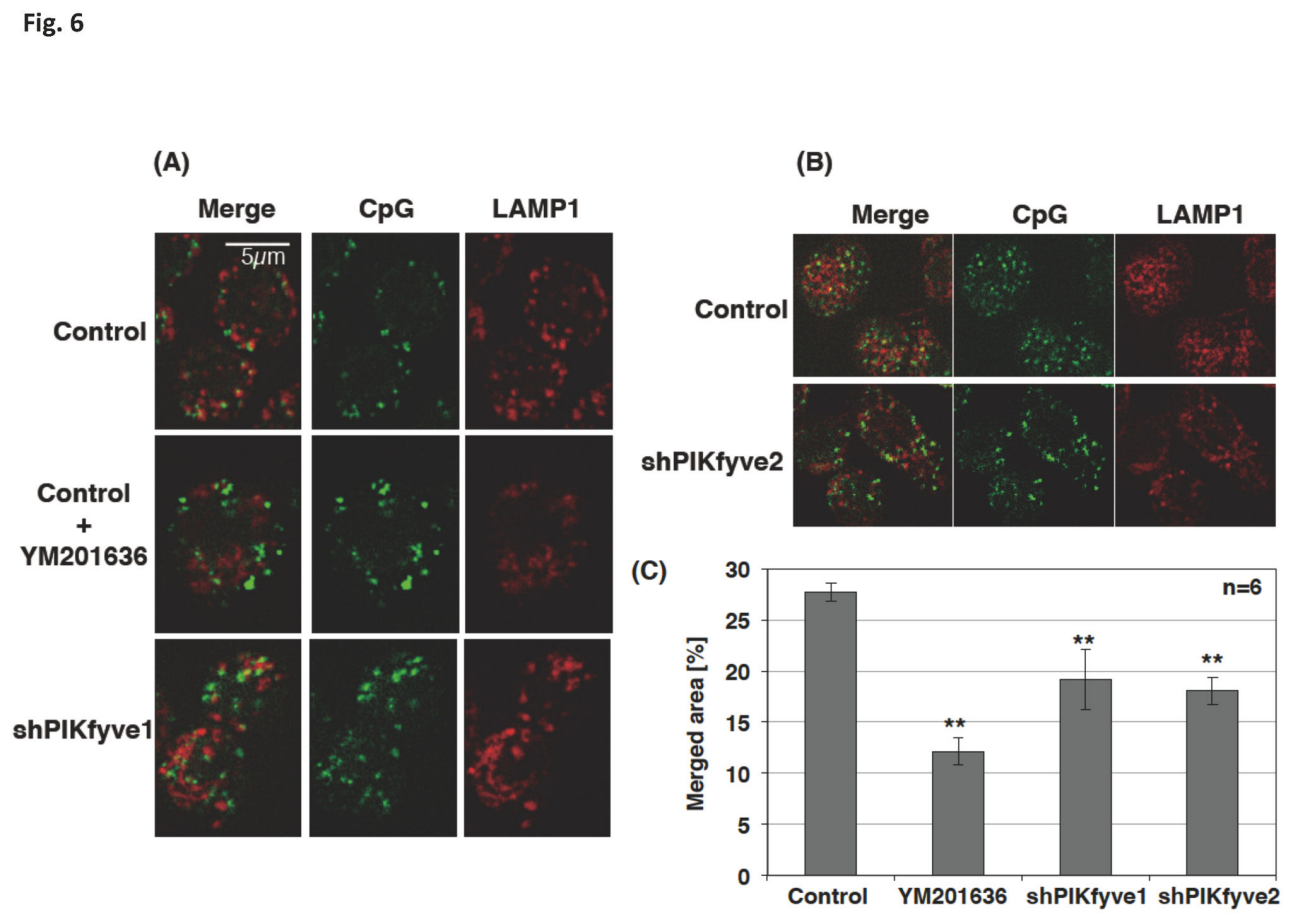
CpG translocation to the late endosome was inhibited in PIKfyve-deficient cells. Control cells, cells treated with 1 µM YM201636 for 30 min, or PIKfyve-deficient cells (shPIKfyve-1 or shPIKfyve-2 cells) were incubated with 0.5 µM FITC-CpG for 10 min. The cells were washed, fixed, permeabilized, and stained with anti-LAMP1 antibody and Alexa 647-labeled secondary antibody. Z-stacks were captured at 1 µm steps over a Z-axis distance of 5 µm. (A, B) Stacked images are shown. (C) The images were analyzed by a quantification software (BZ-H2C). We analyzed 6 stacked images; each contained 250-300 cells, to to estimate the merged area. ** p<0.01.

As expected, the CpG co-localization with the early endosome marker EEA1 was increased in PIKfyve-deficient cells (shPIKfyve-1 and shPIKfyve-2 cells; Figure 5A, B and C). The co-localization of CpG with LAMP1 decreased significantly in these cells (Figure 6A, B and C). The results indicated that PIKfyve contributes to the maturation process of CpG-containing endosomes.

### The inhibition of PIKfyve altered CpG-induced intracellular signaling

**Figure 7:**
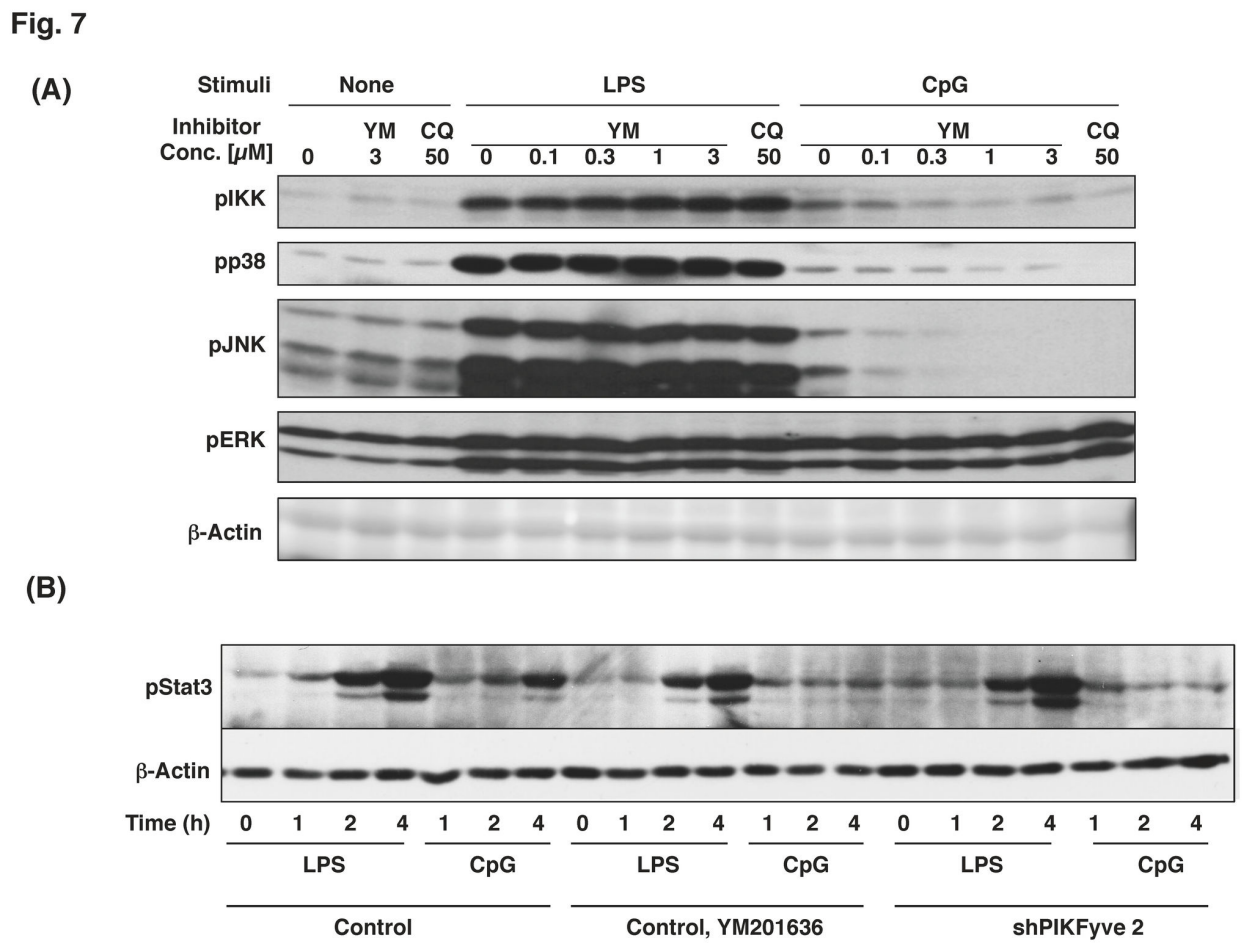
CpG-induced phosphorylation but not LPS-induced phosphorylation of signaling molecules was inhibited by YM201636. (A) Raw264.7 cells were treated with an increasing concentration of YM201636 (YM) or 50 µM chloroquine (CQ) for 15 min. The cells were then stimulated with 20 ng/ml LPS for 15 min or 0.2 µM CpG for 30 min. Total cell lysates were subjected to Western blotting analysis. (B) Control cells, cells treated with 1 µM YM201636 for 15 min, or cells deficient in PIKfyve (shPIKfyve) were stimulated with 20 ng/ml LPS or 0.2 µM CpG for the time indicated. Western blotting analysis was used to detect pStat3.

Stimulation of TLR results in phosphorylation of various signaling molecules. The phosphorylation of IKK α/b results in activation of a transcription factor NF-κB, while the phosphorylation of MAPK cascades leads to activation of AP-1 [5]. Upstream signaling responsible for the NF-κB and MAPK activation bifurcates at the earlier stage [5]. When cells were treated with YM201636 15 min prior to the stimulation, CpG-induced phosphorylation of IKK, p38 MAPK and JNK was severely inhibited in a dose-dependent manner (Figure 7A). In contrast, LPS-induced phosphorylation of p38 MAPK and JNK was not affected by the inhibitor (Figure 7A). LPS-induced IKK phosphorylation was slightly increased in the presence of the inhibitor (Figure 7A). Interestingly, CpG-induced phosphorylation of Erk1/2 was not inhibited by YM201636 (Figure 7A), which suggested that it is not dependent on the endosomal localization of CpG. Chloroquine, which inhibits endosome acidification [21], showed similar effects to YM201636 on the TLR-induced phosphorylation of signaling molecules (Figure 7A). CpG-induced Stat3 phosphorylation but not LPS-induced Stat3 phosphorylation, was inhibited by both YM201636 and by the knockdown of PIKfyve (Figure 7B). The Stat3 phosphorylation induced by these TLRs might be a secondary response induced by TLR4- or TLR9-mediated production of cytokines, such as IL-10, because the event was observed 2-4 h after stimulation with the TLR ligands (Figure 7B).

**Figure 8:**
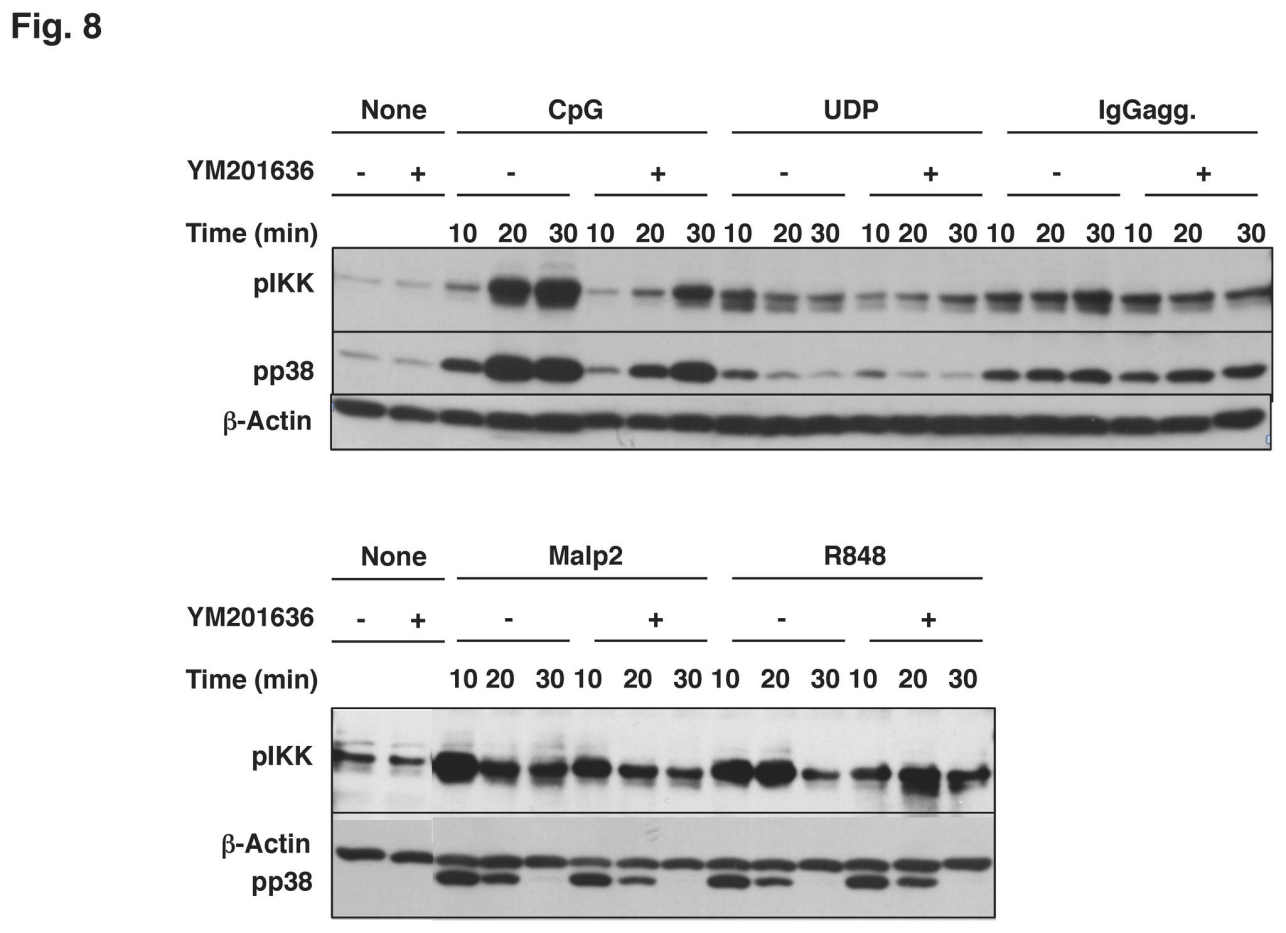
Phosphorylation of IKK and p38 induced by various stimuli was altered in YM201636-treated cells. Raw264.7 cells were treated with (+) or without (-) YM201636 for 15 min. The cells were then stimulated with 1 µM CpG, 0.1 mM UDP, 25 µg/ml IgG (aggregated by incubation at 62 °C, 30 min), 0.2 µM Malp2 or 10 µg/ml R848 for the indicated times. Total cell lysates were subjected to Western blotting analysis.

TLR7, like TLR9, is known to reside in the intracellular compartments. Thus, the signaling events caused by TLR7 ligand may also be inhibited by YM201636. As expected, R848-induced phosphorylation of IKK was inhibited by the PIKfyve inhibitor, although that of p38 was not (Figure 8). PIKfyve may participate in the intracellular trafficking process in different manners, depending on the processes involved in internalization and degradation of the receptor/ligand complex. Although the IKK phosphorylation by Malp2, the ligand of a membrane receptor TLR2, was inhibited by YM201636, the inhibition was not very prominent (Figure 8). The phosphorylation of IKK and p38MAPK by aggregated IgG, which acts via Fcγ receptor, was not susceptible to YM201636 (Figure 8). UDP activates macrophages through a Gi-coupled receptor P2Y. Unexpectedly, the Gi-mediated phosphorylation of IKK and p38 was inhibited by YM201636 (Figure 8). Although further experiments are necessary for underlying mechanisms, it is intriguing to consider that the effect is based on the roles of PIKfyve in translocation of GRCR or arrestin.

### The knockdown of PIKfyve altered CpG-induced intracellular signaling

**Figure 9:**
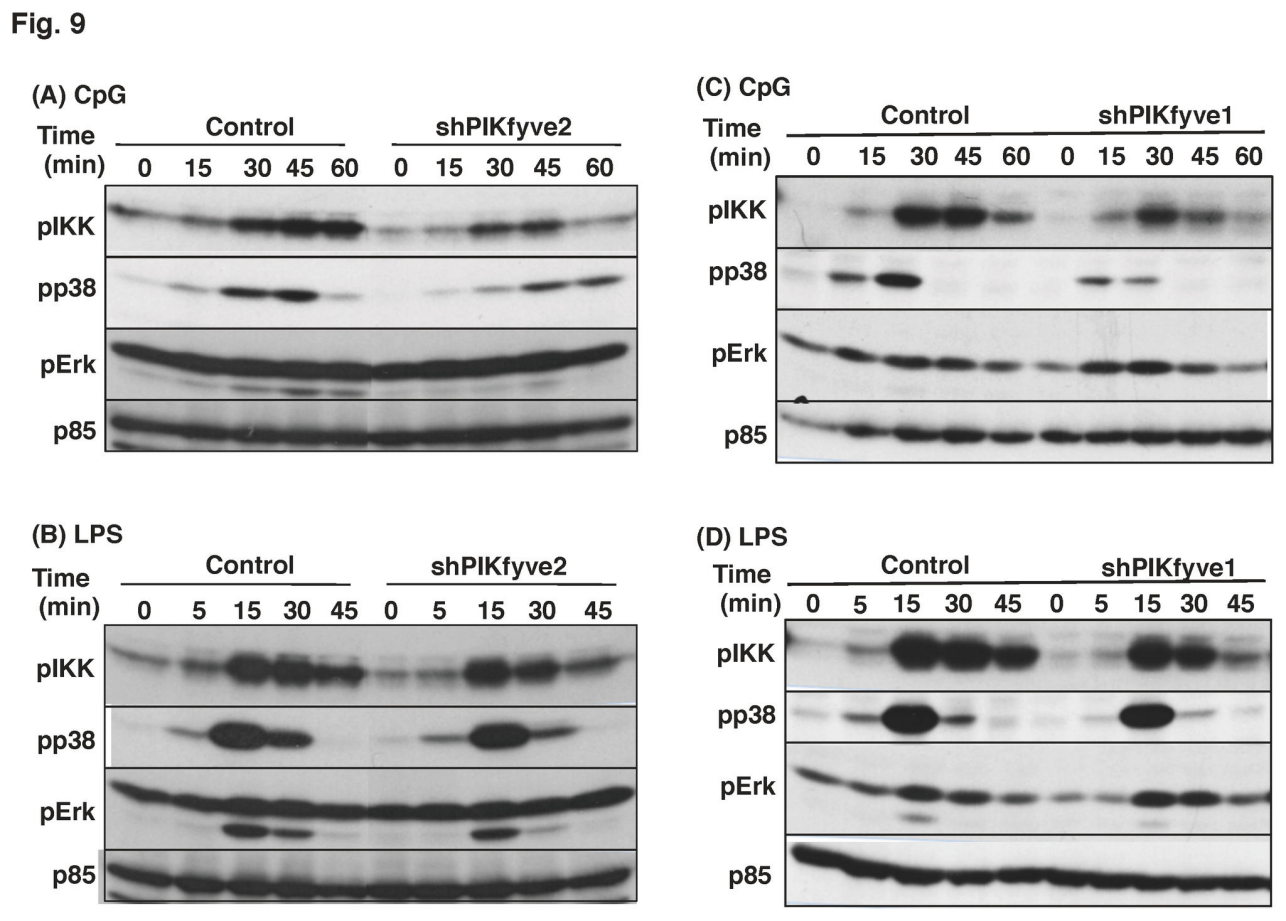
CpG-induced phosphorylation but not LPS-induced phosphorylation of signaling molecules was inhibited by knockdown of PIKfyve. Control or PIKfyve-deficien cells (shPIKfyve-1 or shPIKfyve-2 cells) were stimulated with 0.5 µM CpG (A, C) or 20 ng/ml LPS (B, D) for the time indicated. Total cell lysates were subjected to Western blotting analysis.

As expected, the knockdown of PIKfyve changed the phosphorylation states of the signaling molecules in a manner similar to YM201636 (Figure 9). CpG-induced, but not LPS-induced, phosphorylation of IKK and p38 was decreased by PIKfyve deficiency. Interestingly, CpG-induced phosphorylation of Erk1/2 was almost unchanged in both the knockdown cells (Figure 9) and the YM201636-treated cells (Figures 7, 9).

### The inhibition of PIKfyve altered TLR-mediated cytokine mRNA expression in Raw264.7 cells

**Figure 10:**
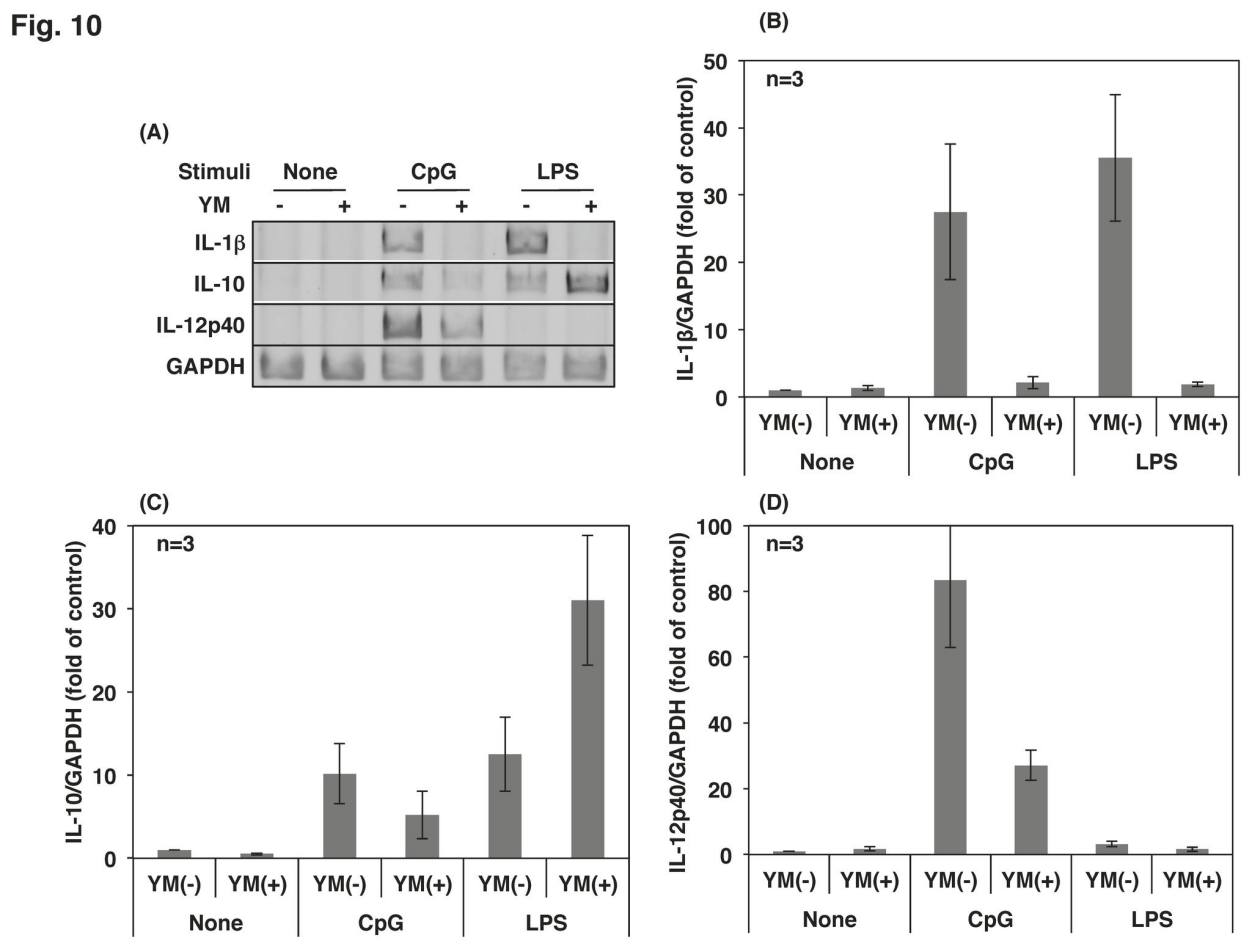
Cytokine mRNA expression was altered by YM201636. (A) Raw264.7 cells were treated with 1 µM YM201636 (YM) for 15 min followed by the addition of 10 ng/mL LPS or 0.5 µM CpG for 4 h. Total RNA was isolated and cDNA was prepared by reverse transcription. The cDNAs of cytokines were amplified by PCR. (B) (C), (D) The results in (A) were quantified by NIH image analysis and calculated as relative intensities to that of GAPDH. The final values on the graph were calculated as the fold of the relative intensity of the control cells. The results from the three separate experiments are shown as the mean ± SD.

**Figure 11:**
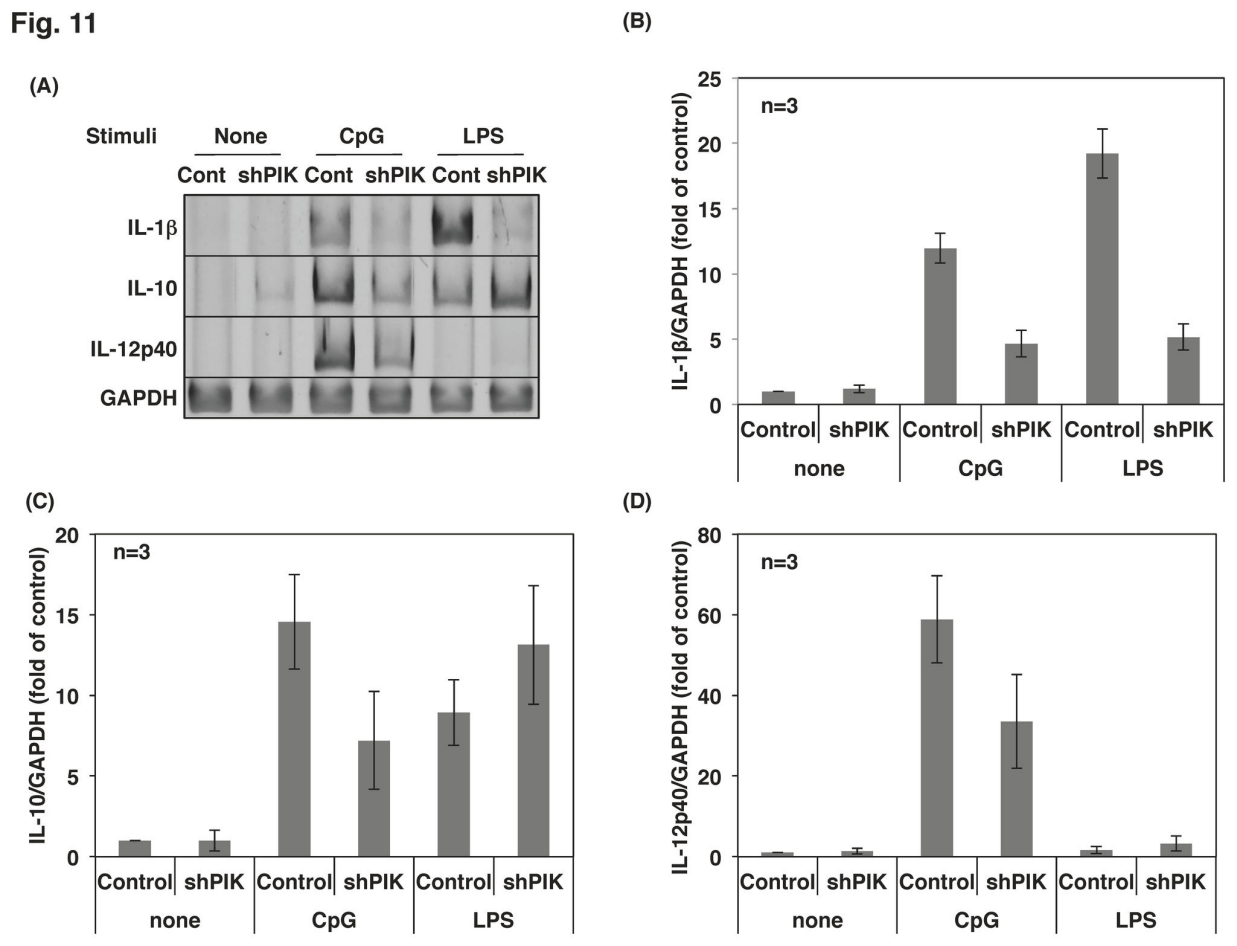
Cytokine mRNA expression was altered by knockdown of PIKfyve. (A) Control (Cont in A) or PIKfyve-deficient cells (shPIK) were stimulated with 10 ng/mL LPS or 0.5 µM CpG for 4 h. Total RNA was isolated and cDNA was prepared by reverse transcription. The cDNAs of cytokines were amplified by PCR. (B) (C), (D) The results in (A) were quantified as described in Figure 10. The results from the three separate experiments are shown as the mean ± SD.

TLR-mediated activation of signaling molecules leads to mRNA expression in macrophages. CpG-induced mRNA expression of IL-1β, IL-12 and IL-10 was severely attenuated by both the PIKfyve inhibitor (Figure 10) and by PIKfyve knockdown (Figure 11). Interestingly, YM201636 decreased LPS-induced expression of IL-1β and increased LPS-induced expression of IL-10 (Figure 10). The effects of the inhibitor are due to PIKfyve inhibition because similar changes were observed in the PIKfyve-deficient cells (Figure 11). A recent report demonstrated that LPS activates TLR4/MyD88/Mal at the plasma membrane and then translocates to the endosome with TLR4 to activate TRAM/TRIF [1,6]. Thus, it is intriguing to consider that LPS activates a signaling pathway that is responsible for IL-10 expression at the plasma membrane/early endosome and for IL-1β expression at the late endosome. LPS-induced expression of IL-12 mRNA was hardly detectable after a 4-h stimulation in Raw264.7 cells. The effect of YM201636 was not determined because longer incubation with the compound exhibited severe cell toxicity. In PIKfyve-deficient cells, LPS-induced expression of IL-12 mRNA was slightly increased (Figure 11D).

### The inhibition of PIKfyve altered TLR-mediated cytokine production in mouse peritoneal macrophages

**Figure 12:**
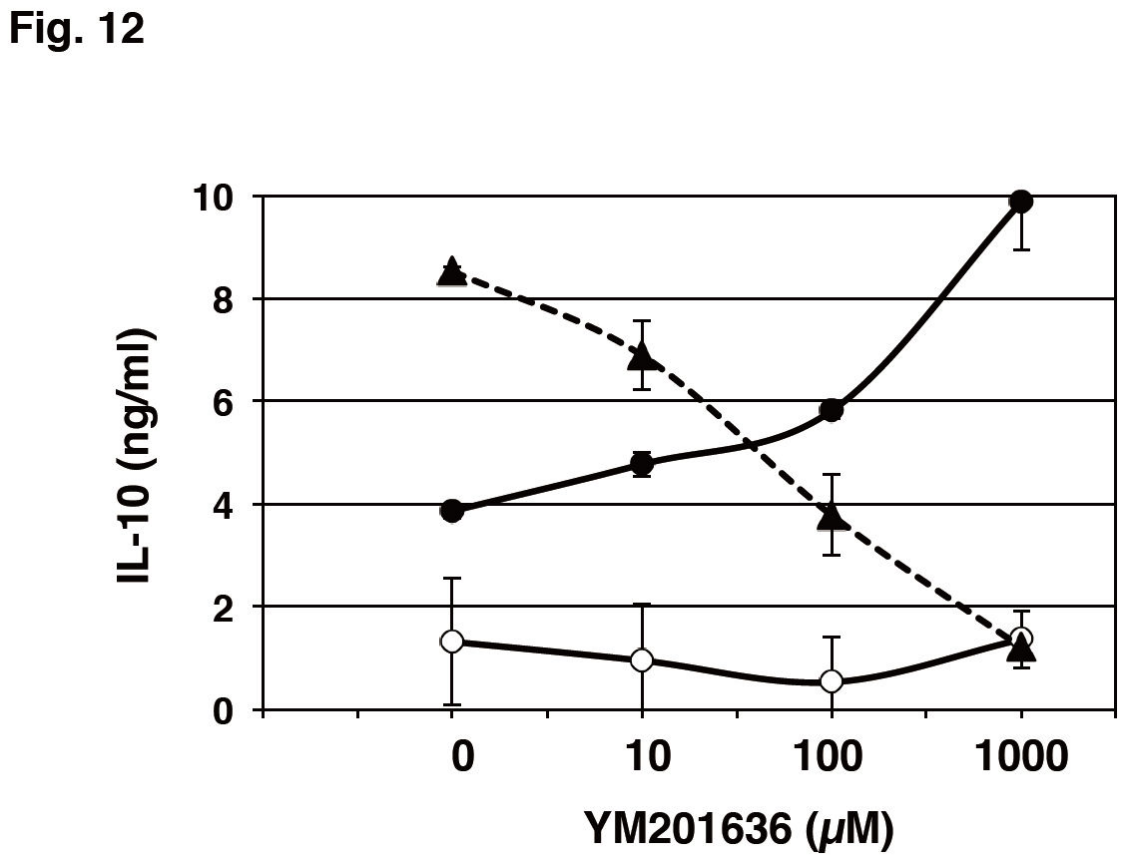
YM201636 decreased IL-10 production induced by CpG but increased that induced by LPS in mouse peritoneal macrophages. Macrophages were treated with an increasing concentration of YM201636 for 15 min followed by the addition of 10 ng/mL LPS (●), 0.5 µM CpG (▲) or vehicle (○) for 16 h. Levels of IL-10 present in the culture medium were determined by ELISA. The results from the three separate mice are shown as the mean ± SD.

**Figure 13:**
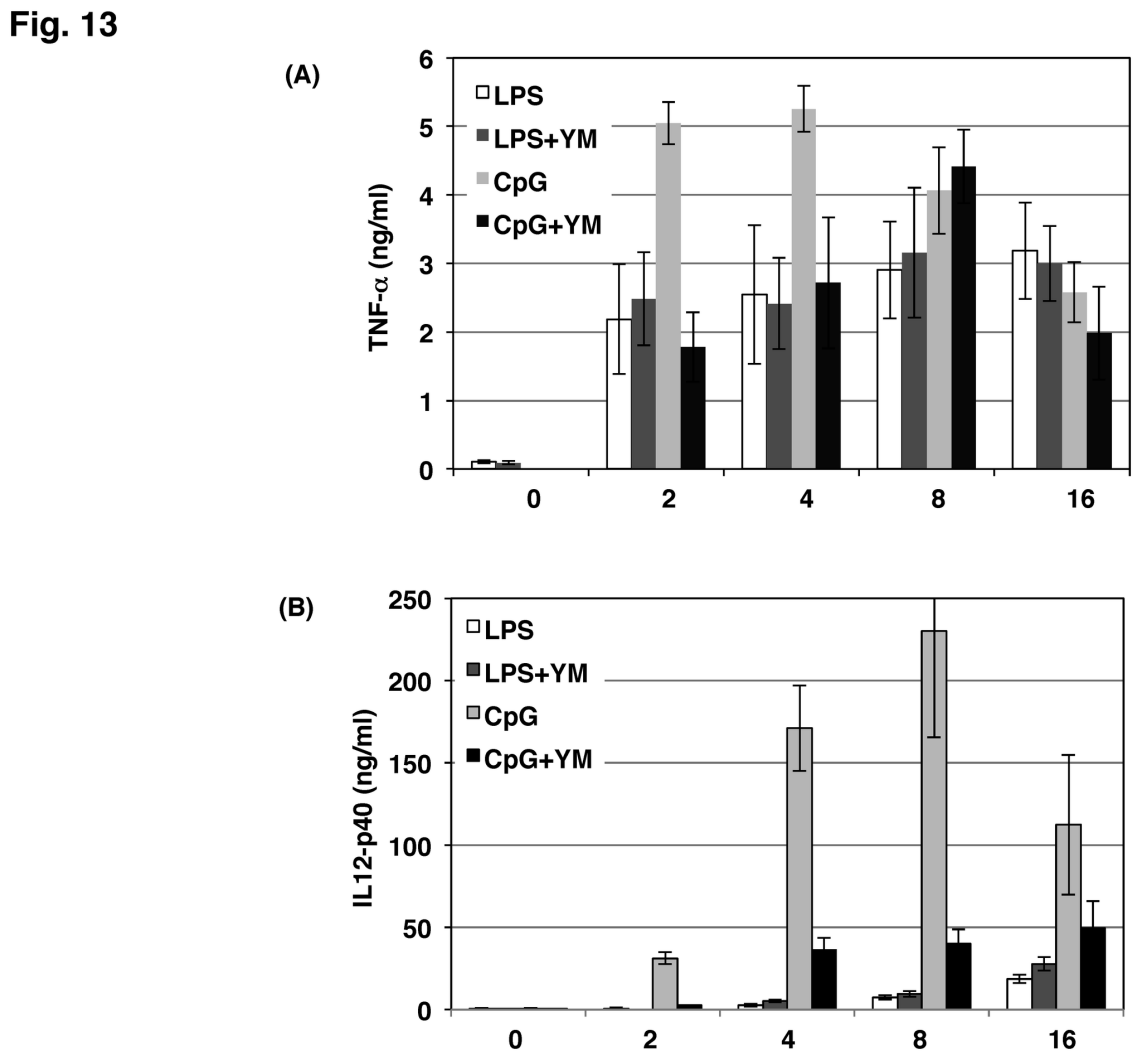
YM201636 influences TNF-α and IL-12p40 production in mouse peritoneal macrophages. Macrophages were treated with 1 µM YM201636 (YM) for 15 min followed by the addition of 100 ng/mL LPS or 1 µM CpG for the time indicated on the abscissa. Levels of TNF-α (A) and IL-12p40 (B) present in the culture medium were determined by ELISA. The results from the three separate mice are shown as the mean ± SD.

The effect of PIKfyve inhibition on IL-10 protein production was examined in mouse peritoneal macrophages (Figure 12). YM201636 inhibited CpG-induced IL-10 production in a dose-dependent manner. In contrast, LPS-induced IL-10 production was increased by the PIKfyve inhibitor. We also determined TNF-α and IL-12p40 production in these cells. CpG-induced production of TNF-α was significantly decreased in the presence of YM201636 at 2-4 h while LPS-induced one was not affected over 16 h (Figure 13A). IL-12p40 production induced by CpG was far greater than that induced by LPS (Figure 13B). YM201636 dramatically inhibited the CpG-induced production of IL-12p40. LPS-induced IL-12p40 production was marginally increased by YM201636. Thus, PIKfyve was considered to regulate cytokine production of mouse macrophages in a manner similar to that seen in Raw264.7 cells.

## Discussion

Much evidence has shown that PIKfyve plays a substantial role in membrane fusion and fission events [22]; however, the role of PIKfyve in TLR-responses has not been reported. In the present study, we demonstrated that PIKfyve is indispensable in the maturation of CpG-containing endosomes, an important step in the TLR9 signaling. The PIKfyve inhibitor YM201636 inhibited the co-localization of internalized CpG with LysoTracker (Figure 3) and prevented CpG from co-localizing with TLR9 (Figure 2). In addition, the maturation of the CpG-containing endosome was disrupted by both the knockdown of PIKfyve and the inhibitor treatment (Figures 4-6). The impaired maturation of CpG-containing endosome was accompanied by the inhibition of TLR9-mediated phosphorylation of signaling molecules (Figures 7-9), which resulted in decreased cytokine mRNA expression and ultimately inhibited cytokine production (Figures 10-13).

We observed that inhibition of PIKfyve activity influences the CpG localization to acidic compartments. The acidification of the CpG-containing endosome can be caused by i) de novo acidification, where the V-ATPase pumps H^+^ into the CpG-endosomes prior to lysosome fusion and ii) lysosome fusion-dependent acidification, where by fusing with lysosomes, existing protons are dumped into CpG-endosomes. Thus, PIKfyve is expected to influence the endosome acidification by either or both mechanisms. In order to clarify whether PIKfyve inhibition impairs the de novo acidification, we have tried to chase this process, but have not succeeded. Further experiments to distinguish two processes are necessary.

Increasing evidence has revealed that both the formation and the maturation of CpG-containing endosomes are critical for the initiation of TLR9-mediated signaling [5,6]. PIKfyve is essential for endosome trafficking, and the finding that PIKfyve knockdown cells failed to respond to CpG supports this conception. Intriguingly, CpG-induced phosphorylation of Erk1/2 occurred normally in both the knockdown cells and in the YM201636-treated cells (Figures 7, 9). In plasmacytoid dendritic cells, the CpG in the early endosome is associated with IFN production, whereas the CpG in the lysosome is associated with NF-κB activation [23–25]. Similarly, it is likely that CpG activates Erk1/2 in the early endosome, whereas it activates IKK, p38 MAPK and JNK in the endolysosome.

TLR4 is a well-characterized cell surface TLR that recognizes LPS with the help of some other proteins, such as LBP, CD14 and MD2 [26]. Upon ligation of TLR4 with LPS, signaling events are initiated through the binding of MyD88 and Mal to the TIR domain of TLR4 [6]. TLR4/LPS then internalizes to the endosome in a dynamin- and clathrin-dependent manner [27]. Because inhibitors of dynamin increased LPS-induced NF-κB activation, the translocation of TLR4/LPS to the endosome had been regarded as a negative regulatory mechanism of TLR4 signaling. Recently, however, another group has revealed that inhibition of TLR4 internalization in macrophages results in a loss of LPS-induced IRF3 phosphorylation without affecting NF-κB activation [28]. Thus, although TLR4/LPS moves to the endosome to be subjected to degradation, TLR4/LPS activates the TRAM/TRIF pathway to produce type 1 IFN before degradation. Gram-negative bacteria-induced IL-1β production is another TRIF-dependent response [29]. Activation of the NLRP3 inflammasome via TRIF ligation is important for the lysosomal degradation of pro-IL-1β [29]. In the present study, LPS-induced IL-1β mRNA expression was severely downregulated by both PIKfyve knockdown and YM201636 treatment (Figures 10, 11), which indicated that the maturation of the TLR4/LPS-containing endosome is indispensable for IL-1β mRNA expression. In agreement with our data, chloroquine, an inhibitor of endosome acidification, decreases LPS-induced IL-1β mRNA expression in human peripheral blood mononuclear cell and macrophages/monocytes [30]. Hence, it is likely that both the mRNA expression and the processing of pro-IL-1β depend on endosome maturation and acidification.

It has been apparent that subcellular localization and trafficking of TLRs and their ligands determine the mode of innate immunity [1]; however, not much is known about the mechanisms that underlie this regulation. Because it has been generally accepted that the key molecules in intracellular trafficking are phosphoinositides [31], we are preparing a series of cells that are deficient in each of the lipid kinases and phosphatases responsible for phospholipid metabolism to investigate their roles in innate immunity.

## Supporting Information

Figure S1 **The sequential focal plane images along with Z-axis.** Raw264.7 cells were incubated with 50 nM LysoTracker Red for 30 min, added with 3 µM FITC-CpG, incubated for additional 10 min and washed. Z-stacks were captured at 0.5 µm steps over a Z-axis distance of 4 µm. In E, the stacked image was constructed.
(TIFF)

Figure S2 **YM201636 did not inhibit PI3-kinase up to 1 µM.** Recombinant p85α/p110β or p85α/p110β were prepared with HEK293T cells, and purified with the antibody against p85. PI3-kinase activities were determined in the presence of increasing concentration of YM201636 or 50 nM wortmannin (Wort) as phosphatidylinositol and [γ-^32^P] ATP as the substrates.
(TIFF)
