# Supplementary figures and images for: PIKfyve Regulates the Endosomal Localization of CpG Oligodeoxynucleotides to Elicit TLR9-Dependent Cellular Responses
Source: PLoS One. 2013 Sep 9;8(9):e73894. doi: 10.1371/journal.pone.0073894 (PMC3767827; doi:10.1371/journal.pone.0073894)

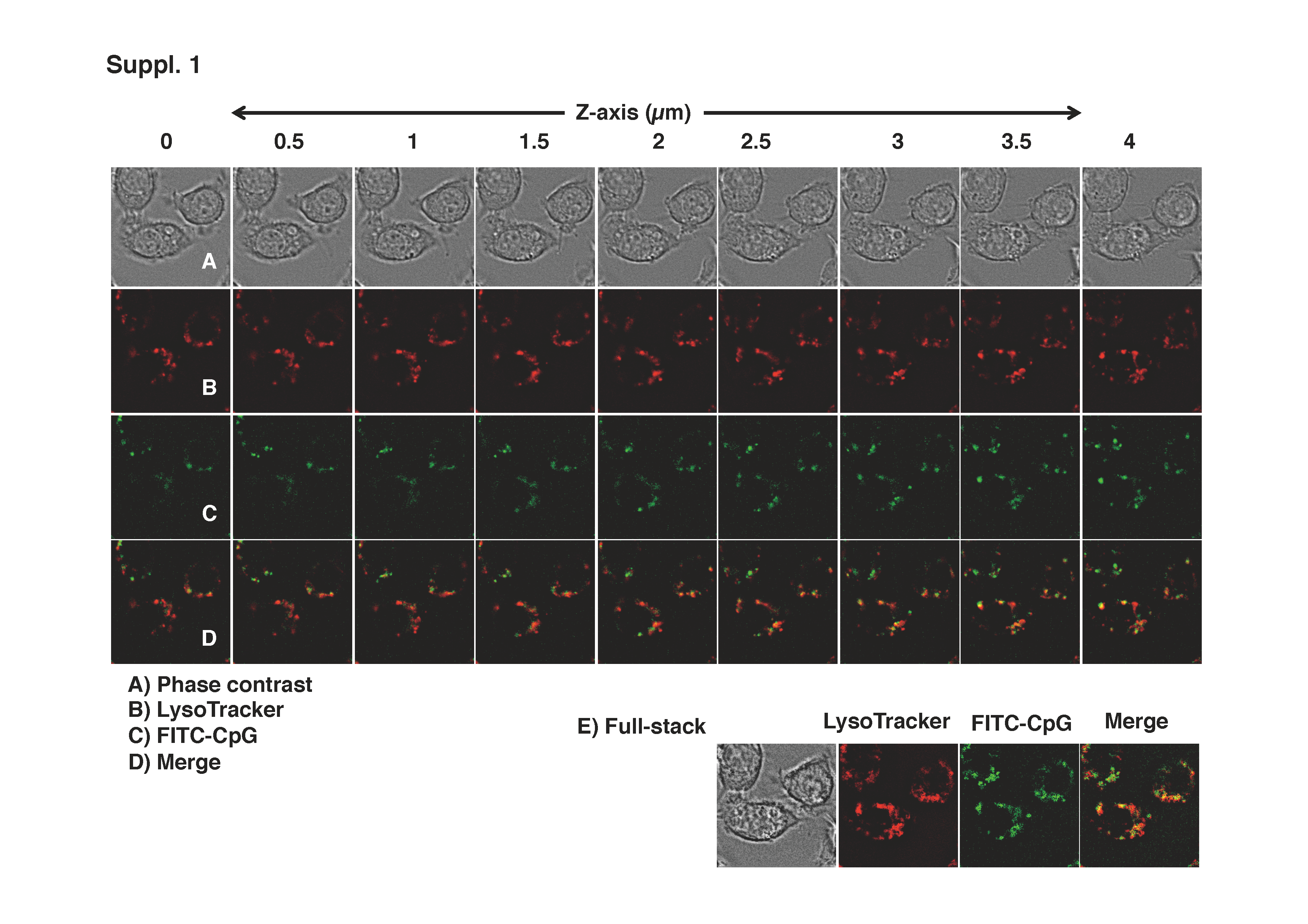

Supplement: Figure S1 — The sequential focal plane images along with Z-axis. Raw264.7 cells were incubated with 50 nM LysoTracker Red for 30 min, added with 3 µM FITC-CpG, incubated for additional 10 min and washed. Z-stacks were captured at 0.5 µm steps over a Z-axis distance of 4 µm. In E, the stacked image was constructed. (TIFF) [file pone.0073894.s001.tiff]

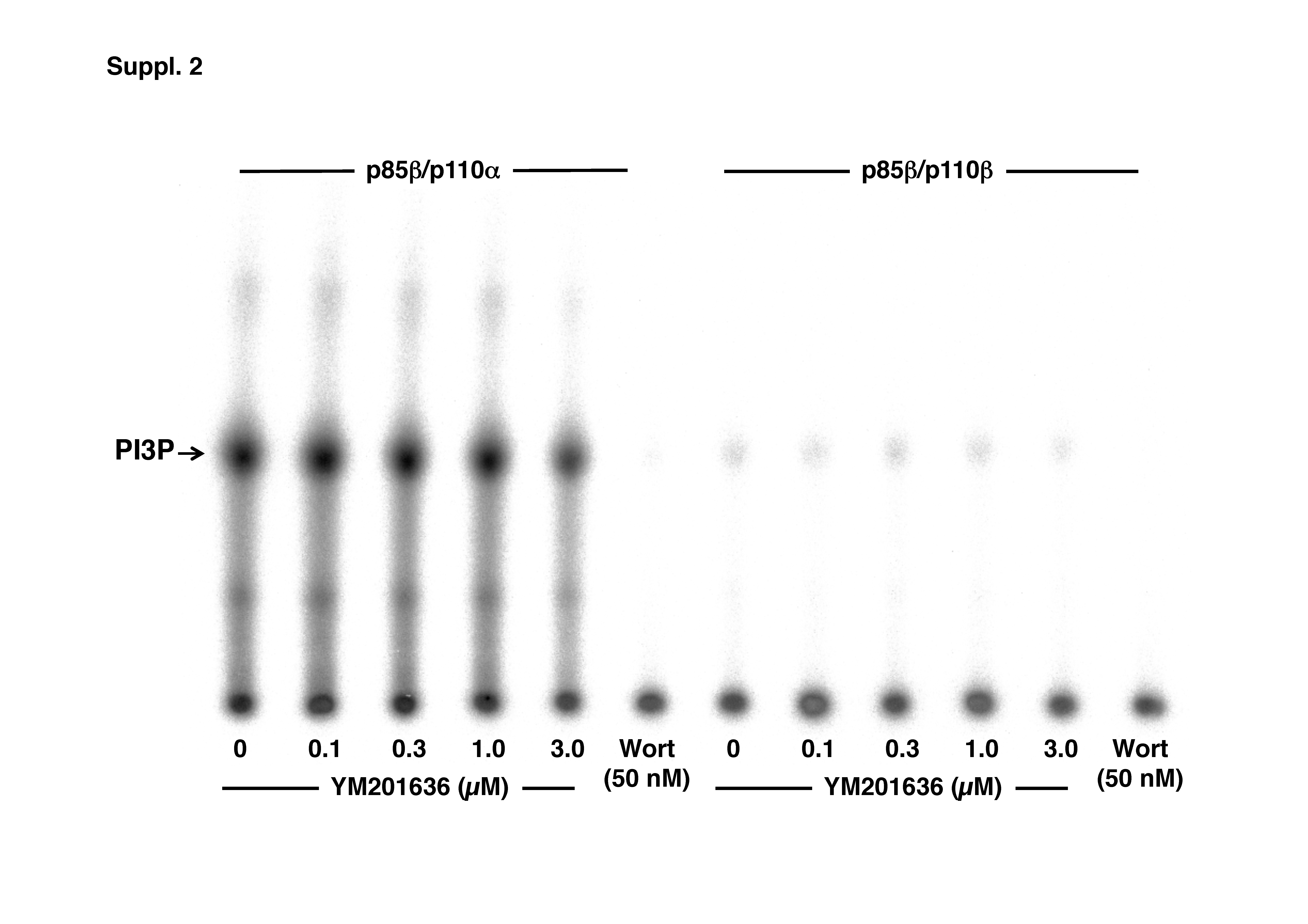

Supplement: Figure S2 — YM201636 did not inhibit PI3-kinase up to 1 µM. Recombinant p85α/p110β or p85α/p110β were prepared with HEK293T cells, and purified with the antibody against p85. PI3-kinase activities were determined in the presence of increasing concentration of YM201636 or 50 nM wortmannin (Wort) as phosphatidylinositol and [γ-32P] ATP as the substrates. (TIFF) [file pone.0073894.s002.tiff]
